# What will it take for an injectable ARV to change the face of the HIV epidemic in high‐prevalence countries? Considerations regarding drug costs and operations

**DOI:** 10.1002/jia2.26106

**Published:** 2023-07-13

**Authors:** Gesine Meyer‐Rath, Lise Jamieson, Yogan Pillay

**Affiliations:** ^1^ Health Economics and Epidemiology Research Office (HE^2^RO), Faculty of Health Sciences University of the Witwatersrand Johannesburg South Africa; ^2^ Department of Global Health Boston University School of Public Health Boston Massachusetts USA; ^3^ The South African Department of Science and Innovation/National Research Foundation Centre of Excellence in Epidemiological Modelling and Analysis (SACEMA) Stellenbosch University Stellenbosch Republic of South Africa; ^4^ Department of Global Health Stellenbosch University Stellenbosch South Africa

**Keywords:** cabotegravir, LAED, PrEP, South Africa, cost, demand/supply

## Abstract

**Introduction:**

The proven effectiveness of injectable cabotegravir (CAB‐LA) is higher than that of any other HIV prevention intervention ever trialled or implemented, surpassing medical male circumcision, condoms and combination antiretroviral treatment. Based on our own analyses and experience with the South African oral pre‐exposure prophylaxis (PrEP) programme, we review the supply and demand side factors that would need to be in place for a successful rollout of CAB‐LA, and delineate lessons for the launch of other long‐acting and extended delivery (LAED) antiretroviral drugs.

**Discussion:**

On the supply side, CAB‐LA will have to be offered at a price that makes the drug affordable and cost‐effective to low‐ and middle‐income countries, especially those with high HIV prevalence. An important factor in lowering prices is a guaranteed market volume, which in turn necessitates the involvement of large funders, such as PEPFAR and the Global Fund, and a fairly rapid scale‐up of the drug. Such a scale‐up would have to involve speedy regulatory approval and WHO pre‐qualification, swift integration of CAB‐LA into national guidelines and planning for large enough manufacturing capacity, including the enabling of local manufacture. On the demand side, existing demand for HIV prevention products has to be harnessed and additional demand created, which will be aided by designing CAB‐LA programmes at the primary healthcare or community level, and involving non‐traditional outlets, such as private pharmacies and doctors’ practices.

**Conclusions:**

CAB‐LA could be the game changer for HIV prevention that we have been hoping for, and serve as a useful pilot for other LAEDs. A successful rollout would involve building markets of a guaranteed size; lowering the drug's price to a level possibly below the cost of production, while also lowering the cost of production altogether; harnessing, creating and sustaining demand for the product over the long term, wherever possible, in national programmes rather than single demonstration sites; and establishing and maintaining manufacturing capacity and supply chains. For this, all parties have to work together—including originator and generic manufacturers, donor organizations and other large funders, and the governments of low‐ and middle‐income countries, in particular those with high HIV prevalence.

## 1 INTRODUCTION

Could injectable cabotegravir (CAB‐LA) be the game changer for HIV prevention that the world has been waiting for—especially low‐ and middle‐income countries (LMICs) with high HIV prevalence? And what can it teach us as we await the launch of other long‐acting and extended delivery (LAED) antiretroviral preparations? CAB‐LA certainly has proven the effectiveness that is higher than that of any other HIV prevention intervention ever trialled or implemented [1, 2], surpassing medical male circumcision [3, 4] and condoms [5]—at least at the current levels of use‐ and even higher than the prevention effect of combination antiretroviral treatment [6]. In a recent analysis for South Africa, home to a fifth of the world's people living with HIV where an estimated 200,000 additional people acquire HIV every year, we showed that, depending on uptake and duration of use, CAB‐LA could prevent between 15% and 28% of HIV infections over 20 years—more than any other single intervention that we have evaluated over the 7 years that we spent re‐optimizing the country's HIV programme through annual Investment Cases [7, 8]. Our findings confirm those of earlier analyses done before the full effect of CAB‐LA was known [9, 10, 11]. What is important to note is that this large reduction in HIV infections would play out over a baseline of the fairly successful existing South African HIV programme, in which all other prevention interventions are already routinely offered, and the first and third UNAIDS 90‐90‐90 targets have been met [12]. CAB‐LA, however, comes at a significantly high price, with the only currently listed prices available being US$4,434,024 per person per year in the United States [13], and US$3801.79 in the UK [14], which point to affordability being a major factor to consider. Against this backdrop, we are painfully aware that for the exceptional benefits to materialize, CAB‐LA would first need to be affordable for an LMIC, such as South Africa.

What else would be needed to be in place for a successful CAB‐LA rollout? As economists and policy analysts, we think about access as the result of demand and supply side factors playing out—if possible, in a coordinated fashion. As members of the South African Department of Health's PrEP technical working group, our views are also informed by the lessons learned while advising on South Africa's oral PrEP programme.

## 2 DISCUSSION

On the supply side, much attention has focussed on the price at which CAB‐LA will be offered to LMIC, including South Africa, by the manufacturer of the originator drug or the three generic manufacturers that have recently been issued with voluntary licenses under a deal brokered by the Medicines Patent Pool (MPP) [15]. This price will define whether CAB‐LA will be affordable to enough governments of high‐burden countries to have a chance at making an impact. By the same token, the more countries that are enabled to rollout CAB‐LA, the lower the price level should become. Very simply put, the total profit that can be generated by a drug (or any other commodity) is a function of the price per unit and the total volume of units sold. (Putting to one side the argument that drugs preventing severe disease should not be a commodity, or that the manufacturer, judging by its own policy guidance documents, “does not expect to profit from sales of its marketed products to public HIV programmes and international donor agency programmes in all low‐income countries, least developed countries, and sub‐Saharan African countries” [16].) This means that a profit can be either made from setting a higher price level—or, just as well, by enabling a larger number of clients to access the drug. History shows how well the latter can work: the same logic was employed to drive down the cost of antiretrovirals in the early 2000s to levels that allowed HIV treatment to be rolled out globally—through enabling generic competition and market shaping. This large volume was in part guaranteed by the involvement of PEPFAR, the Global Fund (GF), and, later, UNITAID—which, we argue, might need to play the same role in getting CAB‐LA to the people who most need it.

But what does this lower price level need to be? The lively current discussion between the manufacturer and international organizations has resulted in estimates of a feasible minimum price of CAB‐LA for HIV programmes ranging from US$16 (excluding capital expenditure) to US$270 per patient per year—assuming 6–7 injections per client per year (for more details on these estimates, see [8]). For South Africa, using the principles of threshold analysis, we established that CAB‐LA would need to cost less than US$105 per patient‐year to be at least as cost‐effective as current oral PrEP [8]. When confronted with these findings, the manufacturer responded by pointing out that this price “significantly underestimates the cost of manufacture,” which “is more expensive and much harder than for generic oral PrEP, which is a simple white tablet” [17]. If this were true, there would indeed be an argument for the involvement of a large donor to bring the price below cost, and to a level acceptable to the South African government, essentially shifting the risk of investment in a novel intervention with as yet unclear demand away from an LMIC government, many of which are experiencing significant economic challenges. Successful examples of this approach are the buy‐down such as that brokered by the Bill & Melinda Gates Foundation for HIV self‐test kits which single‐handedly created the market for millions of self‐test kits to be used by LMIC governments [18]. This would then, in a second step, enable the South African government, which funds the lion's share of the world's largest HIV programme, to commit to a large CAB‐LA rollout programme and, in turn, drive down the cost for the rest of the world—something that it has done successfully for laboratory commodities, such as the MTB Rif Xpert cartridge [19] and HIV viral load tests [20]. If such commitments cannot be made, the second‐best option would be for CAB‐LA to be sold at a price as close as possible to the cost of production—for all LMICs, including middle‐income countries outside of Africa, which are currently not included in the MPP deal but which would stand to benefit substantially [21].

South Africa's HIV budget for the next 3 years contains a sizeable budget for oral PrEP which could be re‐purposed for injectable PrEP at a rate controlled by CAB‐LA's final market price. For a volume large enough to substantially lower prices, however, the pace of a potential CAB‐LA scale‐up is crucial—as it is for the drug's benefits to be as large as estimated by us and others. This requires three elements to fall into place relatively soon: rapid regulatory approval in those countries where the drug has been filed for approval, including South Africa, Australia, Botswana, Brazil, Kenya and Malawi (following the example of Zimbabwe [22] and Uganda [23] where it has already been approved), and a speedy WHO pre‐qualification which is relevant for countries without their own medicines regulatory authority; swift integration of CAB‐LA into national guidelines, where appropriate, and inclusion into PEPFAR and GF drugs lists; and rapidly upscaling large enough manufacturing capacity, including the enabling of local manufacture (another commitment by the manufacturer [24]), and supporting in‐country supply chain management. And while the pace of scale‐up has to be gauged against concerns, such as increased INSTI resistance if routine HIV testing cannot be kept up, modelling studies suggest that even at high levels of integrase inhibitor resistance, the longer‐term mortality benefits of CAB‐LA are still likely to far outweigh the risks [25].

What needs to happen on the demand side? While we strongly believe that good enough health interventions manage to make a case for themselves, this is not always true for interventions whose main benefit may lie in the future or accrues to other people—COVID‐19 vaccination (and its relatively low uptake even in those LMICs that had some supply) being one strong case in point. In South Africa, strong demand for CAB‐LA has been documented [26]—but might be limited to people already on oral PrEP, most of whom who would much rather access an injection once every 2 months than take pills daily, as well as others keen on adding to or changing away from their current prevention options [26]. Programmes will need thus to both harness existing demand (what we sometimes refer to as prevalent demand) and create additional demand (incident demand), including in people who might not be aware of, or have considered for themselves, PrEP even in its oral form—such as heterosexual men. This demand needs to be created and maintained into the future, learning painful lessons from the experience with oral PrEP on which, in South Africa alone, over 560,000 people have been started on PrEP—but very few return for even just a second month [27]. Finally, a strong case has been made for making CAB‐LA access as easy as possible, based on the excellent experience with community‐based, peer‐led prevention care in parts of the world [28]. For this, CAB‐LA programmes would need to be intentionally designed to be a departure from the current oral PrEP programme. In contrast to oral PrEP, CAB‐LA would need to be rolled out at the primary healthcare or community level, involving the lowest staff cadre (potentially necessitating injection into the deltoid instead of gluteal muscle) and simplified monitoring. Extending the dosing interval, as is currently being discussed for both CAB‐LA and other potential LAED products, will further support community rollout, by reducing the number of required injections and, thus, visits to/from a qualified healthcare provider. Additionally, non‐traditional outlets, such as private pharmacies and doctors’ practices, might have to be involved wherever they have a chance of bringing the product closer to clients, and at all of these outlets (including primary healthcare clinics and community programmes), conversations about PrEP initiation would need to be linked to any kind of HIV testing services, as soon as there is a negative test result.

We would be remiss if we did not mention the role of the many planned demonstration and implementation science projects. While we acknowledge that they allow everyone—donors, governments and manufacturer alike—to move ahead with *something* while awaiting cheaper prices, regulatory approval, demand to be created and one of the other parties to commit first, we believe that they could be detrimental to overall progress—by dampening enthusiasm on both the demand and supply side. Arguably, this is something that happened with oral PrEP rollout in South Africa, where an incremental approach to rolling out to successive target populations defined by HIV risk and dependence on small‐scale demonstration projects funded by donors might have inhibited government's commitment to creating, and sustainably funding, a large enough programme to make a difference. (We speculate that it might also have thwarted demand in the targeted population groups who might have read government's hesitancy as proof that there was something wrong with the product proper, and the early focus on key populations might have worked to stigmatize the intervention altogether.) Additionally, the manufacturer's policy of making LMICs’ access to CAB‐LA contingent on their approval of implementation science protocols could reduce access in the crucial time period until generic versions are registered and available in these countries [29].

It is prudent to note that many of these issues will be the same for other injectable antiretroviral products currently still in the pipeline—whether they will be used for prevention or treatment purposes. CAB‐LA will be a test case for how cheaply sterile injections can be manufactured, and how simply their distribution can be organized. While we think about CAB‐LA programming, we need to keep our eye on that pipeline, ready to pivot to new products as soon as their effectiveness is confirmed.

## 3 CONCLUSIONS

We believe, based on our own and the analytical work of others, that CAB‐LA could be the game changer for HIV prevention that we have been hoping for—and could serve as a useful pilot for other LAEDs. Based on our experience with one country's oral PrEP programme, we know that in order for CAB‐LA to realize its potential, all parties have to work together—that includes originator and generic manufacturers, donor organizations, such as PEPFAR and the GF, other funders, such as the Bill & Melinda Gates Foundation, and the governments of LMICs, in particular those with high HIV prevalence, such as South Africa. The work required to make this a reality is cut out for all of us and involves building markets of a guaranteed size; lowering the drug's price to a level possibly below the cost of production, while also lowering the cost of production altogether; harnessing, creating and sustaining demand for the product over the long term, wherever possible, in national programmes rather than single demonstration sites; and establishing and strengthening manufacturing capacity and supply chains. For now, CAB‐LA, together with maintaining high uptake of HIV testing and treatment, has the singular potential to help end AIDS. We collectively have a single shot to show that we dare not waste this potential.

## COMPETING INTERESTS

The authors declare no competing interests.

## AUTHORS’ CONTRIBUTIONS

First draft: GM‐R and LJ; conceptualization: GM‐R, LJ and YP; editing: YP. All authors have read and approved the final manuscript.

## Data Availability

Data sharing is not applicable to no new data generated.
